# Usefulness of Artificial Membrane, Strat-M^®^, in the Assessment of Drug Permeation from Complex Vehicles in Finite Dose Conditions

**DOI:** 10.3390/pharmaceutics12020173

**Published:** 2020-02-19

**Authors:** Florencio Jr Arce, Narumi Asano, Gerard Lee See, Shoko Itakura, Hiroaki Todo, Kenji Sugibayashi

**Affiliations:** 1 Department of Pharmacy, University of San Carlos, Cebu City 6000, Philippines; florencio_arce@yahoo.com (F.J.A.); glsee@usc.edu.ph (G.L.S.); 2 Graduate School of Pharmaceutical Sciences, Josai University, Saitama 350-0295, Japan; naaanr@gmail.com (N.A.); sitakura@josai.ac.jp (S.I.); ht-todo@josai.ac.jp (H.T.)

**Keywords:** Strat-M^®^, alternative membrane, in vitro skin permeation, complex vehicles, cosmetics

## Abstract

The ban on the use of animals in testing cosmetic products has led to the development of animal-free in vitro methods. Strat-M^®^ is an artificial membrane engineered to mimic human skin and is recommended as a replacement for skin. However, its usefulness in the assessment of the permeation of cosmetics in in-use conditions remains unverified. No data have been published on its comparative performance with the membrane of choice, porcine skin. The comparative permeability characteristics of Strat-M^®^ and porcine skin were investigated using Franz diffusion cells. Caffeine (CF) and rhododendrol (RD) in complex vehicles with varying concentrations of polyols were applied as finite and infinite doses. Good rank orders of permeation from finite dose experiments were observed for RD. High correlations were observed in RD permeation between Strat-M^®^ and porcine skin under finite and infinite dose conditions, whereas only finite dose conditions for CF were associated with good correlations. Permeation from formulations with high polyol content and residual formulations was enhanced due to the disruption of the integrity of the Strat-M^®^ barrier. The usefulness of Strat-M^®^ in the assessment of dermal permeation may be limited to finite dose conditions and not applicable to infinite dose conditions or formulations applied in layers.

## 1. Introduction

Prohibitions on the use of animals in testing finished cosmetic products have been in effect since 2004. With the amendment in regulatory policy, the applications of in vitro methods without the use of animal tissues have gained significant attention as tools for the assessment of skin permeation of cosmetic ingredients. Replacements for skin membranes involving the use of artificial membranes (e.g., Strat-M^®^, silicone membrane) designed to mimic human and animal skin offer a competent alternative to estimate permeation of drugs through skin [1,2,3]. 

Strat-M^®^ is an artificial membrane envisioned as an alternative to animal skin. It was engineered to mimic key structural and chemical features of human skin. This multi-layer artificial membrane possesses a tight top layer coated with a lipid blend resembling the lipid chemistry of the human stratum corneum (SC) and a porous lower layer resembling the epidermis and dermis layers [4]. This membrane possesses equivalency to human skin for the skin permeation of many drugs and claims to have better correlations compared with other biological membranes [5]. In 2015, we reported an assessment of the permeation of several chemicals solubilized in phosphate-buffered saline (PBS, pH 7.4) through Strat-M^®^ using infinite dose models. A good correlation coefficient for the permeation of chemicals was found between the artificial membrane with rat and human skin [6]. Recently, a study on the permeation of nicotine from formulations with binary solvents (water and chemical penetration enhancers) applied as a large finite dose (200 μL/0.64 cm^2^) reported similar findings, where a good correlation between the artificial membrane and human skin was found [4]. Other advantages associated with the Strat-M^®^ membrane are its simplicity of handling, low-cost, and low variability of lot-to-lot quality as opposed to animal-based models.

Cosmetic formulations usually lack additives (e.g., chemical penetration enhancers) that alter skin barrier function and promote percutaneous absorption of low molecular weight ingredients. This positions Strat-M^®^ as a suitable substitute for membranes of biological origin in the assessment of ‘simple’ topical formulations, such as cosmetics. Vehicles used in cosmetics are mostly composed of complex mixtures containing components other than water. Therefore, the effect of the combination of components on the permeation through artificial membranes must be investigated. The use of Strat-M^®^ as an alternative membrane in cosmetics for development, product optimization, regulatory compliance, and safety assessments has been encouraged by several reports [4,5,6,7,8]. However, the actual suitability of this material has not yet been verified in the context of the in-use conditions of cosmetics. Merck Millipore provides a limited list of pure solvents and binary vehicles deemed compatible for use with Strat-M^®^ in the assessment of chemical permeation, but there is a lack of data on polyols, which are common ingredients in cosmetic formulations. 

The usefulness of the Strat-M^®^ artificial membrane in the assessment of the permeation of cosmetic active ingredients from complex formulations in in-use conditions has not been clarified. No published data were found on the comparative performance of Strat-M^®^ regarding the permeation of cosmetic active ingredients through porcine skin, despite the recommendations made. Additionally, the recommended use of porcine skin in the assessment of the permeation of cosmetic active ingredients has not been changed. Assessment of the dermal absorption of cosmetic active ingredients is recommended to be performed using porcine skin, because it closely resembles human skin properties, such as permeability to chemicals, thickness and lipid composition [8]. Being the membrane of choice, it is imperative to understand the similarities and establish equivalency and the relationship between Strat-M^®^ and porcine skin in terms of membrane characteristics (e.g., permeability coefficient, flux, permeation of penetrants) to confirm its applicability in evaluating the permeation of cosmetic active ingredients. The appropriate conduct of in vitro skin permeation studies must encompass dose, and the vehicle/formulation should represent the in-use conditions of the intended cosmetic product. The Scientific Committee in Consumer Safety (SCCS) stipulates the conditions for in vitro dermal absorption studies of cosmetics, where experimental dose or the amount applied during use (i.e., layered application), formulation (e.g., finished cosmetics products, complex vehicles), and barrier integrity must be met. Furthermore, sample application during in vitro experiments should mimic human exposure normally at 10 μL/cm^2^ for liquid formulations [9,10,11,12]. In this study, the design of permeation experiments encompassed finite and infinite dose conditions, layered application, the effect of solvents/complex vehicles, and residual formulations to establish the usefulness of the artificial membrane in assessing the permeation of rhododendrol, a lipophilic molecule, and caffeine, a hydrophilic molecule, as model drugs in simulated in-use conditions. Rhododendrol and caffeine were particularly selected as model drugs since these compounds are widely employed as cosmetic active ingredients. Usefulness and membrane-permeation characteristics were evaluated by comparing these parameters with porcine skin. 

## 2. Materials and Methods 

### 2.1. Materials

Rhododendrol (RD, CAS no. 501-96-2, ≥99%, MW: 166.22 g/mol, logP: 1.49) was a gift from Kanebo Cosmetics, Inc. (Tokyo, Japan). Caffeine (CF, MW: 194.19 g/mol, logP: −0.12), methylparaben, and glycerin were purchased from Fujifilm Wako Pure Chemicals Industries, Ltd. (Osaka, Japan). Sorbitol and 1,3-butylene glycol (BG) were purchased from Tokyo Chemical Industry, Co. Ltd (Tokyo, Japan), and dipropylene glycol (DPG) was purchased from Sigma Aldrich Chimie (Saint-Quentin-Fallavier, France). Strat-M^®^ was purchased from Merck Millipore (Tullagreen, Carrigtwohill, Ireland). Frozen porcine ears were supplied by the Central Institute for Feed and Livestock (JA Zen-Noh, Ibaraki, Japan).

### 2.2. Preparation of Formulations

Aqueous formulations of CF and RD (1% *w*/*v*) were prepared by dissolving a sufficient amount of the drug with purified water in a volumetric flask. The concentration of 1% for RD in water was selected instead of 2% due to its limited solubility. 

For complex vehicle-based formulations, a polyol stock composed of DPG (46.15% *v*/*v*), glycerin (23.08% *v*/*v*), BG (20.51% *v*/*v*), and sorbitol (10.26% *v*/*v*) was first prepared. CF (1% *w*/*v*) formulations with high polyol proportions (50% and 75%) and a simulated residual formulation composed of 100% polyol were derived from the stock. RD (2% *w*/*v*) formulations with low polyol proportion (19.5%), high polyol proportions (40% and 61.8%), residual formulations (90.4% and 100%) were also prepared.

### 2.3. In Vitro Permeation Experiment

Porcine skin was isolated from frozen edible porcine ears kept at −80 °C. The preparation and isolation of full-thickness porcine ear skin was performed in accordance with our previous report [11]. To ensure uniformity, skin from the central dorsal region of the ears was harvested. Before excision, visual inspection was performed to ensure the integrity of the skin. Only intact and damage-free skin was excised. Isolated porcine skin (1200–1300 µm) was set in a vertical-type Franz diffusion cell (effective diffusion area of 1.77 cm^2^). Skin surface temperature throughout the experiment was maintained at 32 °C. The receiver compartment was filled with 6.0 mL of purified water. Prior to the application of doses, the skin was hydrated with purified water (1 mL) for 1 hour. Water was then carefully removed and the skin surface was blotted with a cotton swab to remove excess water. For Strat-M^®^ experiments, the membrane (324.6 ± 4.6 µm) was directly set in to a vertical-type Franz diffusion cell with the polyether sulfone side (shiny top layer) upwards. Hydration was not performed because the membrane does not require such pretreatment prior to use. The same experimental conditions were applied for the artificial membrane experiment. A positive displacement micropipette was used to apply CF and RD formulations either as finite ((non-occluded condition); 17.7 or 35.4 μL/1.77 cm^2^; layered, 17.7 μL and 17.7 μL/1.77 cm^2^) or infinite dose ((occluded condition); 1 mL/1.77 cm^2^). Layered application is a type of finite dose condition wherein formulations are applied repeatedly on the same area of the skin. The applied formulation was spread evenly using the back side of a spatula. Aliquots (500 μL) were withdrawn from the receiver solution at pre-determined time points. Permeation experiments were performed for 8 h.

### 2.4. HPLC Analyses of CF and RD

An aliquot (100 µL) of the RD sample collected at every time point was mixed with an equal volume of internal standard (methylparaben), whereas CF samples were mixed with an equal volume of acetonitrile. Samples were then centrifuged at 4 °C for 5 min. Each sample was analyzed using an HPLC system (Shimadzu Co., Kyoto, Japan) equipped with column (Inertsil^®^ ODS-3 4.6 mm × 150 mm, GL Sciences Inc., Tokyo, Japan), system controller (SCL-10A), pump (LC-20AD), degasser (DGU-20A_3_), auto–injector (SIL-20A), column oven (CTO-20A), UV detector (SPD-20A), and analysis software (LC Solution). The column was maintained at 40 °C with the flow rate of the mobile phase at 1.0 mL/min. The mobile phase for RD was acetonitrile and water (25/75, *v*/*v*), and 0.1% phosphoric acid and acetonitrile (10/90, *v*/*v*) was used for CF. Detection of RD and CF was made at 280 and 254 nm, respectively. 

### 2.5. Measurement of Membrane Electrical Impedance

Strat-M^®^ (25 mm) discs were mounted in a vertical-type Franz diffusion cell, identically to the conditions described above. Hydration was not performed prior to the measurement of impedance. PBS pH 7.4 was loaded into the donor and receiver cells. Impedance was first determined for untreated Strat-M^®^ discs using an impedance meter (10 Hz AC, Asahi Techno Lab., Ltd., Yokohama, Japan). The Strat-M^®^ membrane was carefully blotted dry from the donor side, and 10 μL/cm^2^ of polyol stock was applied. After 10 min, polyol was removed from the membrane surface and fresh PBS pH 7.4 was added, and impedance was determined again. 

### 2.6. Statistical Analyses

All experimental data were tested for statistical significance (*p* < 0.05) using Student’s *t*-test. Pearson’s correlation coefficient was used to characterize the relationship between the cumulative amounts of the drug that permeated through the porcine skin and Strat-M^®^. All data were expressed as mean with a standard error. 

## 3. Results and Discussion

### 3.1. Permeation of Drugs under In-Use and Finite Dose Conditions

**Figure 1:**
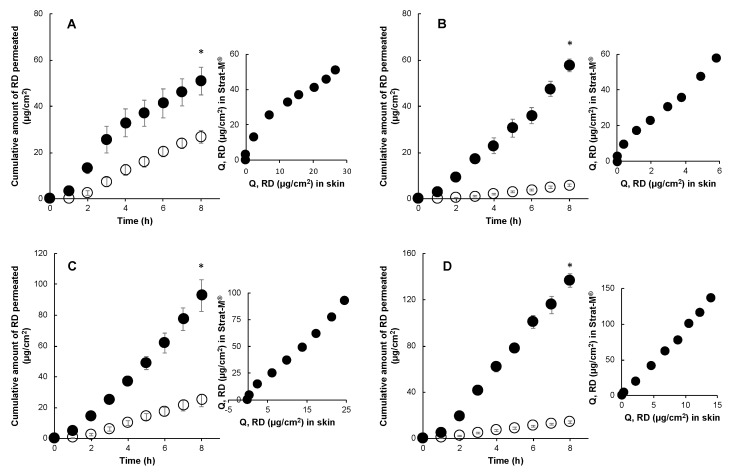
Time course of the cumulative amount of rhododendrol (RD) that permeated through Strat-M^®^ (●) and porcine skin (○) and their correlation under in-use (finite dose) conditions. 1% RD in water single application 10 μL/cm^2^ (**A**), single application 10 μL/cm^2^ lotion (**B**), single application 20 μL/cm^2^ lotion (**C**), layered application 10 and 10 μL/cm^2^ lotion (**D**). Each point represents the mean ± S.E. (*n* = 4). Significant difference (* *p* < 0.05) between RD permeation from single application (10 μL/cm^2^), layered application, single application (20 μL/cm^2^) of lotion, and single application (10 μL/cm^2^) aqueous solution through Strat-M^®^ and porcine skin.

Figure 1 illustrates the cumulative amount of RD that permeated through porcine skin and Strat-M^®^. RD formulations were applied in finite doses (single application of 10 μL/cm^2^, layered application of 10 μL/cm^2^ and 10 μL/cm^2^, and single application of 20 μL/cm^2^) to simulate in-use conditions, such as layered application and dose in human exposures to the liquids [10,11]. Permeation of RD from aqueous and complex vehicle-based formulations through porcine skin showed a dose-dependent rank order except for the layered application, where permeation was lower compared with a single application of 20 μL/cm^2^, despite having the same total applied dose. Water evaporation from the applied formulation in layered application reduced the thermodynamic activity of RD in the residual formulation. RD permeation through the artificial membrane accurately predicted the rank order, as expected, for dose conditions, 10 and 20 μL/cm^2^, whereas the permeation of RD after the layered application was the highest. Strat-M^®^ was able to discriminate between the impact of the applied dose and the composition of the formulation in the permeation of drugs. Excellent correlations (*r^2^* = 0.95–1) existed between permeation through the artificial membrane and skin for RD in all applied dose conditions (Figure 1). 

**Figure 2:**
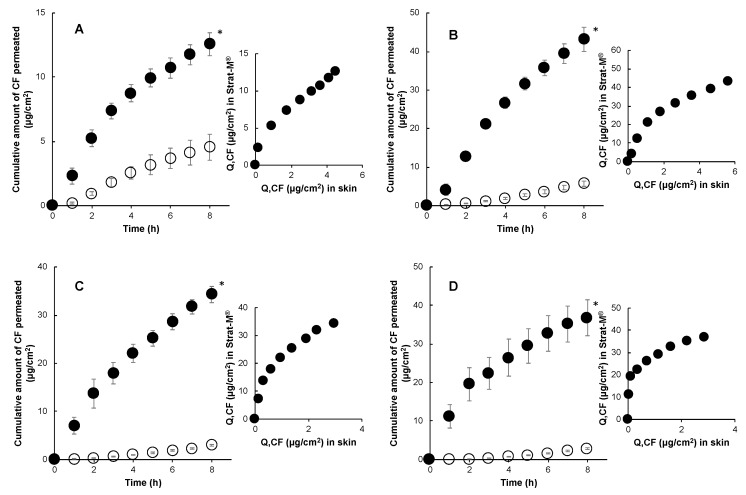
Time course of the cumulative amount of caffeine (CF) that permeated through Strat-M^®^ (●) and porcine skin (○) and their correlations in in-use (finite dose) conditions (10 μL/cm^2^). 1% CF in water (**A**), 1% CF in 50% polyol (**B**), 1% CF in 75% polyol (**C**), and 1% CF in 100% polyol (**D**). Each point represents the mean ± S.E. (*n* = 4). Significant difference (* *p* < 0.05) between RD permeation through Strat-M^®^ and porcine skin.

Permeation experiments through Strat-M^®^ using CF, a hydrophilic model drug, were performed to understand its similarities or dissimilarities with porcine skin. Figure 2 illustrates the cumulative amount of CF that permeated through porcine and Strat-M^®^ from a finite dose application of 10 μL/cm^2^. Permeation of RD and CF from their aqueous formulations through Strat-M^®^ were in good agreement with permeation data through porcine skin (Figure 1 and Figure 2). Fluxes for CF and RD across Strat-M^®^ were in a close range, with minimal enhancement ratios of 1.45 and 1.66, respectively (Table 1). However, finite dose experiments of CF with high polyol proportion and simulated residual formulations revealed significantly higher permeation in contrast to its profile in porcine skin. In skin permeation experiments through porcine skin, CF permeation was enhanced by the formulation containing 50% polyol whereas formulations containing 75% and 100% polyol did not enhance permeation and yielded lower skin permeation, presumably due to diffusional limitations. CF from aqueous formulations yielded the lowest permeation, whereas formulations containing polyol had significantly higher permeation through Strat-M^®^. There was a 4–5.5-fold increase in flux with a corresponding increase in polyol concentration (Table 1). Polyol in formulation apparently enhanced the permeation of CF through the Strat-M^®^ membrane.

### 3.2. Permeation of Drugs under Infinite Dose Conditions

**Figure 3:**
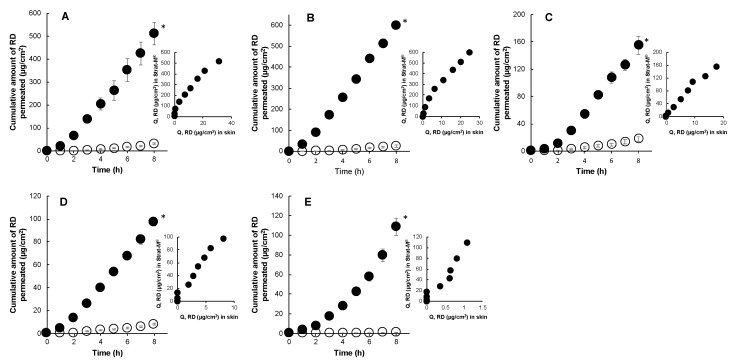
Time course of the cumulative amount of RD that permeated through Strat-M^®^ (●) and porcine skin (○) and their correlations under infinite dose conditions. 1% RD in water (**A**), 2% RD in 19.5% polyol (**B**), 2% RD in 40% polyol (**C**), 2% RD in 61.8% polyol (**D**), and 2% RD in 100% polyol (**E**). Each point represents the mean ± S.E. (*n* = 4). There was a significant difference (* *p* < 0.05) between RD permeation through Strat-M^®^ and porcine skin.

Figure 3 presents the permeation of RD through porcine skin and Strat-M^®^ in infinite dose conditions. RD permeation under finite and infinite dose conditions had identical rank orders of permeation (aqueous formulation (0% polyol) > low polyol concentration (19.55) > high polyol concentration (40% and 61.8%) > residual formulation (100% polyol)) through porcine skin, wherein increasing polyol concentration in formulations corresponds to a decrease in the amount of permeated drug (*r^2^* = 0.98). In this case, the residual formulation was deemed sufficiently viscous to cause diffusional limitations. Permeation enhancement of RD through Strat-M^®^ was observed in the formulation with low polyol concentration (19.5%), whereas the rest of the formulations had similar orders of permeation (Figure 3). This finding suggested similarities in the permeation pathway for RD through porcine ear skin and the artificial membrane. Overall, the artificial membrane demonstrated a high correlation (*r^2^* = 0.94–0.98) of permeation with porcine skin for RD. 

**Figure 4:**
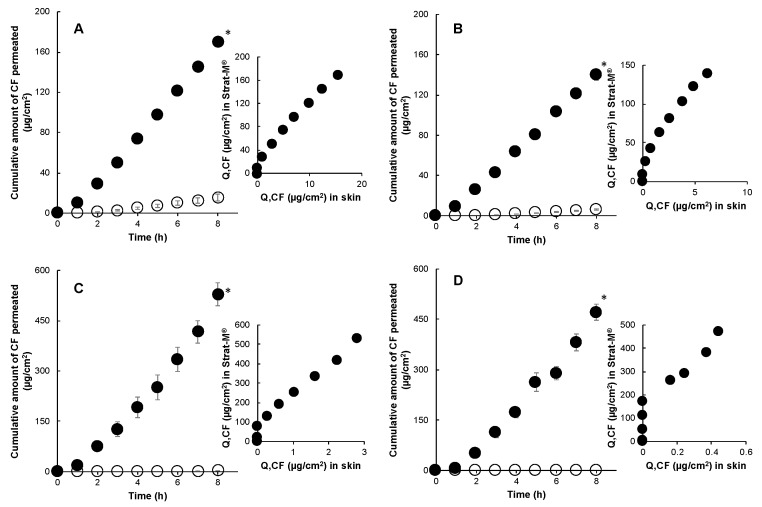
Time course of the cumulative amount of CF that permeated through Strat-M^®^ (●) and porcine skin (○) and their correlations under infinite dose conditions. 1% CF in water (**A**), 1% CF in 50% polyol (**B**), 1% CF in 75% polyol (**C**), and 1% CF in 100% polyol (**D**). Each point represents the mean ± S.E. (*n* = 4). There was a significant difference (* *p* < 0.05) between RD permeation through Strat-M^®^ and porcine skin.

Figure 4 shows the permeation of CF through porcine skin and Strat-M^®^ in infinite dose conditions. CF permeation through skin in infinite dose conditions had an identical rank order (aqueous formulation (0% polyol) > high polyol concentration (50% and 75%) > residual formulation (100% polyol)) of permeation to RD. However, a dissimilar order of permeation for CF was found in permeation experiments through the Strat-M^®^ membrane. The permeation of CF through Strat-M^®^ from formulations with high polyol content and residual formulation was consistently enhanced. This was contradictory to what was observed with skin permeation data, where high proportions of polyol in the residual formulation were found to reduce the thermodynamic activity of CF and RD by solubilization, hence, decreasing the migration of these drugs from polyol to the skin. A lower correlation value (*r^2^* = 0.89) was found between CF permeation through skin and the Strat-M^®^ membrane from its residual formulation, owing to large variations in their concentration–time point profiles (Figure 4D). 

Despite having a relatively high concentration–time point correlation between Strat-M^®^ and porcine skin in most formulations for both drugs, it must be noted that the amount of drug permeating through Strat-M^®^ in all dose conditions was significantly higher than that of skin. Haq and colleagues have presented evidence on the effect of polyol-membrane interaction on drug permeation [4]. The cumulative amount of drug that permeated from their control formulation composed of a pure polyol (propylene glycol) through Strat-M^®^ resulted in a 14-fold increase in permeation. Solubilization or lipid extraction of lipophilic structures on its top layer may have caused similar results, particularly the increase in permeation, despite being applied as finite doses, as observed in both studies. Additionally, the artificial membrane and formulations with high polyol proportions used in this study share lipophilic qualities. Strat-M^®^ lacks the highly organized intercellular structures of the SC, therefore, it simply does not mimic the heterogeneous complexity of the SC and fails to render similar barrier properties to those of the SC to provide the ideal interaction of vehicles with SC lipids [13]. Since the partitioning of a drug into the skin is dependent on its ability to preferentially ‘transfer’ from the formulation into the SC and beyond, this may have been the reason for the unusually high permeation of CF in an infinite dose, as opposed to how it would permeate from a residual formulation through porcine skin. In the porcine skin-based experiment, formulations with high polyol content and residual formulations did not result in an increase in flux. In addition, the diffusivity of chemicals through an artificial membrane is related to its permeation route, with the relatively thin SC-like layer thus providing a low-tortuosity pathway, which is probably the reason for higher permeability of hydrophilic compounds [6].

The permeation ratio of CF and RD permeation through Strat-M^®^ and porcine skin is presented in Table 2. The flux for CF from a formulation of high polyol content (75%) and the residual formulation through Strat-M^®^ was 160- and 739-fold higher, respectively. A low correlation between the permeation enhancement of CF and polyol content through Strat-M^®^ exists. However, it is notable that higher fluxes can be observed in CF formulations containing high polyol content (75% and 100% polyol). Moreover, the permeability coefficient of Strat-M^®^ was enhanced proportionally with increasing concentration of polyol which is probably a concentration-dependent disruption of Strat-M^®^’s barrier integrity. Hence, an increase in the permeability coefficient can be observed with higher polyol content. Flux of CF through Strat-M^®^ is proportionally enhanced in formulations of high polyol content as well, where applied. Reduction in flux was observed in porcine skin-based experiments where high polyol content could reduce the thermodynamic activity of the permeating drugs. A correct prediction for RD flux was obtained from its aqueous and high polyol formulations. RD, in its residual formulation, showed 121-fold higher permeation through Strat-M^®^. This finding was also observed when we conducted identical experiments using another residual formulation containing 90.4% polyol, and it was found that Strat-M^®^ was more permeable (133-fold higher) when formulations with very high polyol content were applied. 

Permeability coefficients were elevated in formulations with high polyol content and residual formulations. No relationship exists between the permeability coefficients of porcine skin and Strat-M^®^. The permeability coefficients of CF through Strat-M^®^ increased proportionally with the amount of polyol in the formulation, whereas the opposite was observed in experiments using porcine skin with both drugs (Table 2). In RD, a good rank order for aqueous and high polyol formulations (40% and 61.8%) was seen between porcine skin and Strat-M^®^, however, significant enhancement in permeability was seen in a formulation with low polyol content (19.5%). 

### 3.3. Effect of Polyols on the Drug Permeation Through Strat-M^®^

We assessed the impact of commonly used polyols as vehicles/solvents in cosmetics on the drug permeation through Strat-M^®^ in in-use conditions. The electrical impedance of the membrane confirms the integrity of the membrane’s barrier property. The compositions of the formulations in this study represent common solvents used in many cosmetic formulations, as well as their formulation dynamics after being applied onto the skin. The electrical impedance of the membrane was found to be high (≥100 kΩ·cm^2^), indicating the good barrier properties of its SC-like top layer. Application of polyols, typical solvents in cosmetic formulations, to Strat-M^®^ for 10 min resulted to a significant reduction (92%) in impedance (post-treatment impedance value of 8.02 ± 0.89 kΩ·cm^2^) of the membrane. The application of polyol-based formulations appeared to solubilize the lipid-based SC-like top layer of the artificial membrane. In layered applications, the application of the first layer disrupts the barrier integrity. Hence, it promotes higher RD permeation through Strat-M^®^ from the second layer applied, as opposed to the known lowering of permeation through porcine skin in layered applications. This also supported the unusually high permeation of CF with high polyol and residual formulations in both finite and infinite dose systems through Strat-M^®^. 

**Figure 5:**
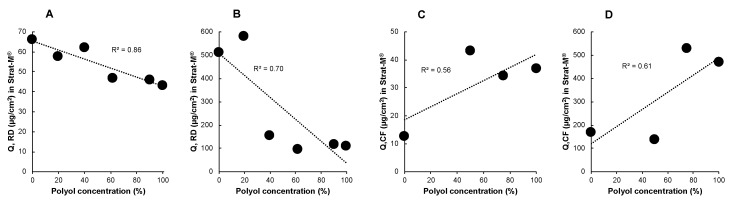
Relationship between permeation through Strat-M^®^ and polyol concentration in RD and CF formulations. Finite dose experiment with RD (**A**), infinite dose experiment with RD (**B**), finite dose experiment with CF (**C**), and infinite dose experiment with CF (**D**).

The impact of high amounts of polyol remaining on the skin has been established to markedly reduce the permeation of cosmetic active ingredients, and a poor correlation existed between polyol content and permeation. Permeation of RD through Strat-M^®^ from a range of polyol concentrations was inversely correlated with polyol concentrations in the formulation for finite and infinite dose conditions with *r*^2^ values of 0.86 and 0.70, respectively. RD permeation through Strat-M^®^ is not correlated with polyol concentration in the formulation because the disruption of the barrier integrity is likely to be limited to the lipid-based top layer of the artificial membrane. The hydrophilic viable epidermis-like layer of Strat-M^®^ must have remained intact throughout the experiment and, thus, effectively limited the passage of the lipophilic RD molecule. In the case of CF, despite having lower correlations between the cumulative amount of permeation and polyol concentration in finite (*r*^2^ = 0.56) and infinite dose experiments (*r*^2^ = 0.61) (Figure 5), an enhanced permeation through Strat-M^®^ was observed. Moreover, high permeation of CF through Strat-M^®^ under finite and infinite dose conditions was exhibited by formulations with high polyol content. Enhanced permeation of hydrophilic drugs, such as CF, has been previously reported in membranes with reduced electrical impedance due to compromised barrier function [14,15]. 

## 4. Conclusions

High correlations (*r^2^* = 0.94–1) in permeation between Strat-M^®^ and porcine skin under finite and infinite dose conditions were observed with RD, whereas these were only observed in finite dose conditions for CF. A poor relationship was obtained between the permeability coefficients of CF and RD through Strat-M^®^. The amounts of RD and CF that permeated through Strat-M^®^ from complex vehicles was higher in both dose conditions. Similar permeability characteristics between the two membranes can be observed from aqueous formulations. 

Permeation of drugs from formulations with high polyol content and residual formulations was increased with an increase in the permeability of the artificial membrane. The barrier integrity of Strat-M^®^ was breached upon contact with high concentrations of polyol by lipid extraction or solubilization of its SC-like top layer, as indicated by the drastic reduction in electrical impedance. The use of Strat-M^®^ in the assessment of dermal permeation of cosmetics may be limited to formulations with low polyol content and finite dose conditions. Assessment of permeation from concurrent application of identical or non-identical formulations (i.e., layered application) and infinite dose conditions with the use of Strat-M^®^ could result in overestimation of the permeation parameters. Good rank order of permeation from formulations with complex vehicle-based formulations applied as finite doses was observed with a lipophilic compound (RD). Findings from this study suggest the selective potential usefulness of artificial membranes in discriminating the effects of complex vehicle formulations and predicting permeation of cosmetic active ingredients. Further assessment of the permeation of cosmetic active ingredients should be performed by employing other solvent systems and formulations to enhance the applicability of Strat-M^®^ in cosmetic formulation design, optimization, and safety assessments. 

## Figures and Tables

**Table 1:** Permeation parameters of CF and RD from various formulations through Strat-M^®^ and porcine skin under finite dose conditions.

Formulations	Strat-M^®^	Porcine Skin	Permeation Ratio (*J*_Strat-M_^®^/*J*_Skin_)	*r*^2^ (*Q*_Strat-M__®_ vs. *Q*_Skin_)
	*J* (μg/cm^2^/h)	*J* (μg/cm^2^/h)		
1% CF in water	0.92 ± 0.05	0.63 ± 0.09	1.45	0.96
1% CF in 50% polyol	5.80 ± 0.27	1.42 ± 0.33	4.07	0.89
1% CF in 75% polyol	2.87 ± 0.27	0.61 ± 0.06	4.73	0.87
1% CF in 100% polyol	3.13 ± 0.53	0.57 ± 0.04	5.47	0.71
1% RD in water	4.08 ± 0.42	2.45 ± 0.12	1.66	0.93

*J*—flux; *Q*—cumulative amount of drug permeated; *r*^2^—correlation coefficient.

**Table 2:** Permeation parameters of CF and RD from various formulations through Strat-M^®^ and porcine skin under infinite dose conditions.

Formulations	Strat-M^®^		Porcine Skin		Permeation Ratio (*J*_Strat-M_^®^/*J*_Skin_)	*r*^2^ (*Q*_Strat-M__®_ vs. *Q*_Skin_)
	*P* (cm/s) × 10^−7^	*J* (μg/cm^2^/h)	*P* (cm/s) × 10^−7^	*J* (μg/cm^2^/h)		
1% CF in water	7.25 ± 0.26	24.01 ± 0.87	1.75 ± 0.36	2.76 ± 0.59	8.71	0.98
1% CF in 50% polyol	4.87 ± 0.17	19.57 ± 0.60	0.36 ± 0.01	1.20 ± 0.15	16.3	0.96
1% CF in 75% polyol	46.6 ± 3.73	93.92 ± 1.66	0.17 ± 0.02	0.59 ± 0.06	160.4	0.97
1% CF in 100% polyol	22.2 ± 1.99	67.97 ± 2.63	0.02 ± 0.005	0.09 ± 0.02	739	0.89
1% RD in water	25.7 ± 1.89	79.99 ± 6.78	6.55 ± 1.75	9.27 ± 0.54	8.63	0.96
2% RD in 19.5% polyol	1,327,853 ± 44,308	95.15 ± 6.92	3.02 ± 1.41	4.60 ± 0.74	20.7	0.97
2% RD in 40% polyol	3.32 ± 0.48	24.39 ± 3.01	0.41 ± 0.07	3.37 ± 0.59	7.24	0.98
2% RD in 61.8% polyol	1.76 ± 0.036	14.53 ± 0.29	0.09 ± 0.03	0.97 ± 0.22	15.03	0.98
2% RD in 100% polyol	3.58 ± 0.22	18.37 ± 2.81	0.01 ± 0.001	0.15 ± 0.01	121	0.94

*P*—permeability coefficient; *J*—flux; *r*^2^—correlation coefficient.
